# Neuroinflammation as a Central Mechanism in Alzheimer’s Disease: Therapeutic Insights from Schiff Base Derivatives

**DOI:** 10.3390/molecules31030465

**Published:** 2026-01-29

**Authors:** Siti Khadijah Abdullah, Wah Seng See-Too, Taznim Begam Mohd Mohidin, Gokula Mohan

**Affiliations:** 1 Institute of Biological Sciences, Faculty of Science, Universiti Malaya, Kuala Lumpur 50603, Malaysia; sma190006@siswa.um.edu.my; 2 Novabio Celltech Sdn Bhd, Lot 56935, Jalan 9/8, Seksyen 9, Bandar Baru Bangi 43650, Malaysia; felix@novabiocelltech.com

**Keywords:** Alzheimer’s disease, neuroinflammation, Schiff bases

## Abstract

Despite decades of intensive research, an effective cure for Alzheimer’s disease (AD) remains elusive. Although AD is classically linked to amyloid-beta (Aβ) aggregation, growing evidence highlights neuroinflammation as a major driver of disease progression. Neuroinflammation forms a self-amplifying cycle involving various factors such as cytokines, chemokines, oxidative stress, and glial cell activation, emphasizing the need for multi-target therapeutic strategies. Schiff bases have emerged as promising candidates, especially metal-incorporated Schiff bases, as numerous preclinical studies have demonstrated their ability to modulate key pathological processes, including inflammation, oxidative stress, reactive oxygen species (ROS) impairment, metal dysregulation, Aβ aggregation, and cholinergic dysfunction. Additionally, some preclinical studies even revealed the neuroprotective and anti-amnesic potential of Schiff bases. Nevertheless, these activities have been investigated across diverse structures of Schiff bases, and systematic evaluation of metal-incorporated Schiff bases remains limited. Although Schiff base-based anti-AD investigations have remained exclusively at the preclinical level, the huperzine A prodrug ZT-1 progressed to early-phase clinical trials before its development was discontinued. Comprehensive studies assessing their multi-target potential with their pharmacokinetic profiles are therefore essential to advance their development as prospective anti-AD agents.

## 1. Introduction

Alzheimer’s disease (AD) is the leading cause of dementia, affecting over 55 million people globally, with numbers expected to nearly double every 20 years [1]. Despite extensive research efforts, current pharmacological interventions for AD remain primarily symptomatic, offering limited efficacy in halting or reversing disease progression [2]. Traditionally, AD has been attributed to amyloid-beta (Aβ) plaques and neurofibrillary tangles composed of hyperphosphorylated tau protein. However, an increasing body of evidence highlights the significance of neuroinflammation as a pivotal mechanism driving AD pathophysiology [3,4].

Neuroinflammation in AD is largely mediated by the activation of microglia and astrocytes, which recognize aggregated Aβ and cellular debris. These glial cells produce pro-inflammatory cytokines such as interleukin-1β (IL-1β), tumor necrosis factor-alpha (TNF-α), and interleukin-6 (IL-6), contributing to chronic inflammation, synaptic loss, and neuronal death [5]. Importantly, this inflammatory cascade not only exacerbates pathology but may precede and facilitate the deposition of Aβ and tau aggregates.

Given the multifactorial nature of AD, there is growing interest in the development of multi-target therapeutic agents. Interestingly, Schiff bases characterized by the presence of an imine or azomethine (-C=N-) functional group demonstrated promising pharmacological activities relevant to neurodegeneration extensively at the preclinical level. Their anti-inflammatory, antioxidant, reactive oxygen species (ROS) scavenging, metal-chelating, Aβ inhibitory, and anti-cholinesterase properties, as well as their ability to protect the neurons and improve memory, render them valuable scaffolds in the search for effective AD therapeutics [6]. Strikingly, the huperzine A-derived Schiff base prodrug ZT-1 (Debio 9902) progressed to early-phase clinical trials but was not further developed [7,8].

This review aims to discuss the role of neuroinflammation in AD pathogenesis and highlight Schiff bases as multifunctional agents capable of modulating neuroinflammation and treating AD.

## 2. Neuroinflammation as a Central Pathological Mechanism in AD

**Figure 1:**
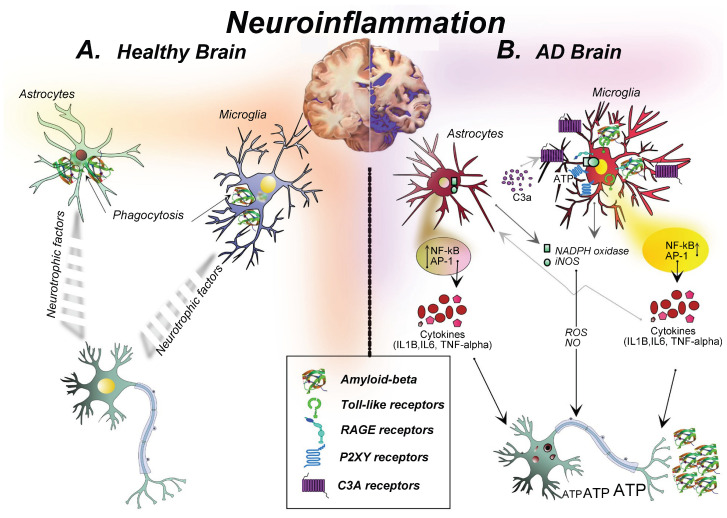
Neuroinflammation in the healthy and AD brain. Reproduced from Ref. [12] under the Creative Commons Attribution 4.0 International License (CC BY 4.0). To view a copy of this license, visit http://creativecommons.org/licenses/by/4.0/ (accessed on 13 December 2025).

AD is a multifactorial disease arising from a complex interplay of genetic risk factors, environmental influences, aging, and lifestyle-related triggers and is widely believed to be driven by Aβ production and deposition. Microglia are the brain’s resident immune cells and act as the first line of defense in response to a pathological event [9]. In the presence of Aβ aggregation, the microglia are activated by the binding of Aβ to its pattern recognition receptors (PRRs) (Figure 1), such as the triggering receptor expressed on myeloid cells 2 (TREM2), Toll-like receptors (TLRs), receptors for advanced glycoxidation end-products (RAGEs), and nucleotide-binding oligomerization domain (NOD)-like receptors (NLRs). The activated microglia, referred to as disease-associated microglia (DAM), undergo morphological and molecular modifications to enhance the production of pro-inflammatory cytokines, generate ROS and reactive nitrogen species (RNS), inhibit phagocytosis, accelerate Aβ plaque accumulation, induce neurotoxicity, promote inflammasome formation, and drive the maturation of IL-1β [10,11,12]. These alterations eventually stimulate amyloidogenesis, hinder the clearance of Aβ, and cause neurotoxicity [13].

Similar to microglia, astrocytes are activated by Aβ aggregation through PRR binding and undergo morphological changes known as cell hypertrophy. The activated astrocytes, referred to as reactive astrocytes, release abundant neurotoxic factors such as ROS, nitric oxide (NO), cytokines, and chemokines [14]. An abundance of neurotoxic factors interrupts the homeostasis of amyloid precursor protein (APP), causing accumulation of Aβ and neurotoxicity leading to the death of neurons and oligodendrocytes [12,14].

The activation of both microglia and astrocytes enhances the production of cytokines, chemokines, and ROS. The upregulation of pro-inflammatory cytokines such as TNF-α, IL-1β, and IL-6 disrupts the synaptic plasticity, impairs long-term potentiation, and induces neuronal apoptosis [5]. On the other hand, chemokines such as CCL2, CCL3, CCL5, and CXCL10 recruit more immune cells to the brain, enhance the accumulation of activated glial cells, and overwhelm the production of neurotoxic factors, causing synaptic dysfunction and cognitive failure [15]. ROS cause lipid oxidation, resulting in oxidative stress and DNA damage, which accelerates the aging and apoptosis of neurons. In addition, ROS alter the redox potential of Aβ-associated metal ions, leading to mitochondrial dysfunction, eventually causing death of the neurons [16]. Collectively, inflammation, oxidative stress, and glial activation form a self-perpetuating cycle that accelerates AD pathology.

## 3. Schiff Bases: Chemistry and Biological Properties

### 3.1. Structural Features and Synthesis

Schiff bases are identified based on the imine (–C=N–) functional group it carries, which can occur naturally or be derived from nature. Most of the known Schiff bases today are synthesized by chemists through a condensation process between a primary amine and an aldehyde or ketone. Hugo Schiff synthesized the first Schiff base in 1864, and the structure was named after him [17]. The chemically active imine bond and the structural modifiability of the precursors (amines and carbonyls) allow precise modifications to achieve optimal physicochemical characteristics like lipophilicity, redox potential, and metal-binding affinity. These physicochemical properties need to be considered to ensure the crossing of the compound across the blood–brain barrier (BBB), overcoming the oxidative stress and Aβ aggregation. Additionally, the Schiff base compounds’ functional readiness with electron-donating or electron-withdrawing groups, heterocycles, and pharmacophores provides a platform for multi-target-directed ligands (MTDLs). Thus, the capability of the compound to serve the requirement and interact with multiple targets makes it an ideal candidate for AD drug development.

### 3.2. General Pharmacological Properties

Over the past two decades, Schiff bases have been increasingly recognized for their multifaceted bioactivity. Numerous in vitro and in vivo studies demonstrate the anti-inflammatory, antioxidant, antifungal, and antibacterial potential of Schiff bases [18,19]. The ability of Schiff bases to form complexes with metal ions such as Cu^2+^, Zn^2+^, and Fe^3+^ is an added advantage, as these ions are found excessively around Aβ plaque. This is due to the loss of homeostasis of these ions, which progressively participate in redox reactions and promote the aggregation of Aβ plaques [20]. Apart from that, Schiff bases have been proven for their potential to inhibit enzymes favorable to AD, namely acetylcholinesterase (AChE) and butyrylcholinesterase (BChE) [21]. These enzymes break down the neurotransmitter acetylcholine, which is essential for learning and memory, thus causing a cholinergic deficit and subsequent cognitive decline. The excellent structural adaptability and pharmacological characteristics of the Schiff bases make them promising candidates as anti-AD agents.

### 3.3. Schiff Base–Metal Complexes

Schiff base–metal complexes often display enhanced biological activity compared to their parent ligands. This is because the Schiff bases act as bidentate or tridentate ligands, which can bind to metal ions at two or three sites, respectively. The formation of Schiff base–metal complexes causes electron delocalization, making it less polar or more lipophilic, thus improving its redox buffering capacity, membrane permeability, and biological half-life [19]. As such, metal complexes often display improved pharmacokinetics and central nervous system (CNS) bioavailability, especially when chelation prevents Fenton-type reactions that propagate ROS generation. In the context of AD, Schiff base–metal complexes have demonstrated better neuroprotective capability compared to the standard drug, Tacrine, and its parent ligand [22]. Furthermore, the inhibition of an AD-favorable enzyme, AChE, was found to be proportional to the number of copper atoms present in Schiff base–metal complexes, proving that the presence of metal ions enhances the therapeutic potential of Schiff bases [23]. Additionally, antioxidant activities studied through lipid peroxidation, free radicals scavenging, and the reduction of ferric and cupric ions involved in Fenton reactions were amplified by the incorporation of metals in Schiff bases [24]. This enhanced anti-AChE and antioxidant potential of Schiff base–metal complexes represents a significant advantage in modulating pathological neuroinflammation in AD.

## 4. Therapeutic Potential of Schiff Bases Against Neuroinflammation in AD

AD is a multifactorial neurodegenerative disease caused by complex interconnections between oxidative stress, neuroinflammation, metal ion dyshomeostasis, Aβ accumulation, and cholinergic dysfunction. Schiff bases have demonstrated various biological properties, including antioxidant, anti-inflammatory, metal-chelating, and enzyme-inhibitory activities (Table 1), making them excellent candidates for multi-target-directed ligands MTDL. The MTDL approach is increasingly recognized as a promising strategy for addressing complex multifactorial neurodegenerative diseases [2]. Moreover, the excellent structural flexibility of Schiff bases allows their modification to optimize biological activities in regulating neurodegeneration. Therefore, Schiff bases possess significant therapeutic potential against neuroinflammation in AD.

### 4.1. Anti-Inflammatory Activity

A growing body of evidence indicates that Schiff base derivatives can significantly regulate key inflammatory mediators. As such, a study reported that the expression of pro-inflammatory genes, TNF-α and IL-6, in a systemic model induced with gastric ulcer is downregulated in Schiff base-treated rodents compared to positive control rodents induced with gastric ulcer [41]. TNF-α is a primary cytokine produced by activated microglia and astrocytes through activation of the nuclear factor kappa-light-chain-enhancer of activated B cells (NF-κB) pathway. Nuclear translocation of NF-κB subsequently drives the transcription of multiple pro-inflammatory genes, including TNF-α itself, IL-6, and other cytokines. Schiff bases disrupt the TNF-α positive feedback loop by inhibiting the translocation of NF-κB factors into the nucleus and exhibit anti-inflammatory activity [42]. TNF-α also plays a central role in initiating and regulating the pro-inflammatory cytokine cascade through other pathways such as the mitogen-activated protein kinase (MAPK) pathway, apoptosis pathway, and Janus kinase/signal transducer and activator of transcription (JAK/STAT) pathway. Similarly, IL-6 activates the MAPK and JAK/STAT pathways. The activation of these pathways enhances Aβ plaque formation, chronic glial activation, and neuronal apoptosis, which leads to an upsurge in the production of neurotoxic factors such as cytokines, chemokines, ROS, RNS, and NO, causing neuroinflammation [5]. Chronic neuroinflammation compromises synaptic function and cognitive performance, leading to the progression of AD. Meanwhile, interleukin-10 (IL-10), an anti-inflammatory cytokine, was significantly upregulated in rodents treated with Schiff bases compared to the positive gastric ulcer control [41]. Activation of the NF-κB pathway halts the expression of anti-inflammatory cytokines; hence, disruption of the TNF-α positive feedback loop can resume the production of anti-inflammatory cytokines. This may potentially assist in restoring homeostasis of neurotoxic factors, thereby preventing neuroinflammation and halting the progression of AD [14]. Thus, Schiff bases can potentially mitigate AD progression.

### 4.2. Antioxidant Activity and ROS Scavenging

The redox-active nature of Schiff bases enables them to function as scavengers and reducing agents of free radicals and metal ions, respectively [34], which eventually lessens the oxidative stress that intensifies inflammation and neuronal damage. Schiff bases exhibit antioxidant and scavenging properties via hydrogen atom transfer (HAT) and single electron transfer (SET) mechanisms [43]. Numerous derivatives, especially those incorporating phenolic, quinonoid, or hydroxyl-rich moieties, have demonstrated high radical-scavenging indices in erythrocytes and *in chemico* studies, often surpassing those of conventional antioxidants such as ascorbic acid [34,44,45,46]. This dual action, encompassing both oxidative and inflammatory suppression, is vital in preventing the progression from early synaptic dysfunction to overt neurodegeneration.

### 4.3. Metal Chelation and Inhibition of Aβ Aggregation

A notable hallmark of AD involves the pathological interaction of metal ions such as Zn^2+^, Cu^2+^, and Fe^2+^ with Aβ peptides, facilitating their aggregation and fostering oxidative damage via Fenton chemistry [20]. Schiff bases, particularly those with oxygen and nitrogen donor atoms, form stable complexes with these metals, sequestering them from pathological reactions. Some Schiff base–metal complexes have shown a remarkable capacity to reduce Aβ fibril formation in the neuroblastoma cell line (SH-SY5Y), as observed through Thioflavin T fluorescence assays and electron microscopy [28,29]. Additionally, the utilization of transgenic CL4176 *Caenorhabditis elegans* (*C. elegans*), which expresses human Aβ at high temperatures, shows that the expression of human Aβ is delayed in Schiff base-treated *C. elegans* [32]. The inhibition or downregulation of Aβ expression represents an effective therapeutic strategy to overcome AD, as Aβ aggregation initiates and drives disease progression.

### 4.4. Anti-Cholinesterase Activity

The inhibition of cholinesterase remains a crucial component in developing an anti-AD drug, as standard drugs employ this strategy. This is because cholinergic dysfunction is one of the earliest and most consistent changes observed in AD pathology. Although standard drugs do not resolve the underlying problem, they mitigate disease progression by inhibiting the responsible enzymes. Remarkably, pyrazole-bearing Schiff bases have exhibited comparable inhibition against AChE as the standard drug donepezil in an *in chemico* study [33]. Meanwhile, the synthesis and evaluation of sixteen novel pyrrole-derived triazole Schiff bases demonstrated that six of the compounds possess better inhibition against AChE and BChE compared to the standard drugs donepezil and allanzanthone in an *in chemico* setup. Molecular docking studies of these compounds suggest that Schiff bases inhibit cholinesterase by engaging in multiple key bindings with the enzymes [21]. The dual inhibition of cholinesterase enzymes by Schiff bases is a promising therapeutic strategy, as BChE activity often compensates for declining AChE levels in later stages of AD.

### 4.5. Neuroprotective Activity

Neuroprotective potential should be prioritized in the development of AD drugs, as disease progression occurs due to Aβ plaque formation, which eventually leads to neuronal degeneration. This neuronal degeneration causes cognitive impairment, dementia, memory loss, and behavioral abnormalities, which increase vulnerability to other complications and ultimately result in the death of AD patients [47]. Hence, the neuroprotective properties exhibited by azo-Schiff bases against the SH-SY5Y neuroblastoma cell line indicate their potential as anti-Alzheimer agents. Cu (II)- and Zn (II)-incorporated azo-Schiff bases are preferable to the parent ligand, as they have exhibited neuroprotective activity equivalent to the standard drug Tacrine [22]. Additionally, apoptosis of SH-SY5Y cells caused by Aβ was significantly reduced by Pt (II) diimine Schiff bases. It is suggested that Schiff bases exert neuroprotective activity by interacting with Aβ, the primary factor implicated in AD [28].

### 4.6. Anti-Amnesic Activity

Irreversible neuronal degeneration causes steady memory loss in AD patients, drastically affecting their daily life and self-reliance [47]. Thus, an anti-AD drug capable of improving memory deficits would offer a meaningful therapeutic advancement in restoring patients’ individuality. The synthesis and investigation of six vanillin Schiff bases for short-term memory or amnesic effects using a Y- maze apparatus in an albino mouse model concluded that two Schiff bases were able to significantly improve memory retention in mice by 80% compared to the standard drug, galantamine. Furthermore, the novel object recognition test (NORT), conducted to investigate the effect of the vanillin Schiff bases in the mice model in recognizing familiar objects, demonstrated the capability of four vanillin Schiff bases to enhance recognition memory. It is deduced that inhibition of cholinesterase enzymes by Schiff bases leads to the conservation of acetylcholine in the brain, thereby improving memory [30].

## 5. Clinical Translation of Schiff Base Derivatives in AD: Lessons from Huperzine a Prodrug ZT-1

(−) Huperzine A is an alkaloid originally extracted from the Chinese club moss, a plant traditionally used to treat blood disorders and conditions such as pyrexia, hypertension, and schizophrenia [8]. Over the decades, huperzine A has been extensively synthesized and studied, leading to the discovery of its potential in inhibiting AChE [8]. However, oral administration of huperzine A is limited by its short half-life and is often associated with adverse effects such as nausea, vomiting, diarrhea, and borborygmus. These side effects are mainly attributed to the inhibition of butyrylcholinesterase (BuChE) in the peripheral nervous system [7]. These limitations led to the development of the prodrug ZT-1 by Debiopharm for the symptomatic treatment of AD. The huperzine A prodrug ZT-1 was synthesized by the reaction between the primary amine of (−) huperzine A and the aldehyde of 5-chloro-o-vanillin to form a Schiff base derivative. The 5-chloro-o-vanillin moiety contributes to stability under biological conditions by reducing the electron density around the imine functional group. Additionally, the formation of an intramolecular six-membered ring due to hydrogen bonding further stabilizes the Schiff base [8].

Preclinical studies revealed ZT-1 as the safest derivative compared to over 100 huperzine A derivatives, as it is less likely to affect the peripheral system. Hence, ZT-1 was selected for a phase I clinical trial involving fifty-eight healthy male volunteers. The phase I clinical trial evaluated the tolerance and pharmacokinetics of single and multiple oral doses of ZT-1. The pharmacokinetics study involving nine volunteers for a single oral dose indicated that ZT-1 was rapidly hydrolyzed and excreted as huperzine A by 30–40%. This is consistent with the preclinical study in mice reporting the rapid observation of ZT-1 in the intestinal tract [48]. Meanwhile, a similar number of volunteers in the multiple-dose study revealed that a steady level of huperzine A was achieved within two days post oral administration, with modest accumulation. For the tolerance study in forty healthy young males, no adverse effects were observed in hematology, urinalysis, or biochemistry tests at 2 and 48 h post-dosing. However, electrocardiogram analysis showed changes post-dosing, resulting in sinus bradycardia in more than 60% of volunteers; the symptoms resolved in 2 h without medical intervention. In the multiple-dose study, one volunteer experienced nausea and vomiting 24 h after the first dose. However, the volunteer completed the study as the effects resolved without any medications. Phase I concluded that ZT-1 was reasonably tolerated in healthy young males, although the electrocardiogram findings require further investigation in subsequent clinical trials [7].

Following that, a phase IIa clinical trial for daily oral dosing and a phase I clinical trial for monthly injectable and biodegradable sustained-release implant ZT-1 were conducted. However, the findings were only reported in Debiopharm press releases rather than in peer-reviewed journal articles. The phase IIa oral administration trial utilized the Alzheimer’s Disease Assessment Scale—Cognitive Subscale (ADAS-cog), the Mini-Mental State Examination (MMSE), and the Neuropsychiatric Inventory Questionnaire (NPI-Q) to evaluate improvements in patients with mild to moderate AD. The phase IIa clinical trial, conducted over 9 months, declared ZT-1 to be safe and effective in treating moderate AD. Meanwhile, the phase I clinical trial for ZT-1 implants concluded that ZT-1 was well-tolerated and had no safety concerns. Thus, the phase II Better Recollection for Alzheimer’s patients with ImplaNts of ZT-1 (BRAINz) study was conducted in AD patients to measure the safety and efficacy of ZT-1 implants [49]. However, the findings were never reported after the 2007 press release.

Therefore, it remains uncertain whether the drawbacks observed in the phase I oral administration were fully addressed, especially related to the electrocardiogram findings. ZT-1 was selected among huperzine A derivatives due to its higher BuChE/AChE IC_50_ ratio [7]. However, this indicates residual BuChE inhibition, which is typically more relevant at later stages of AD. Inhibition of BuChE is known to induce gastrointestinal effects such as nausea and vomiting, which were observed in one volunteer in a phase I trial. The limited sample size of fifty-eight healthy volunteers may have underestimated the true incidence of such effects. Although the pharmacokinetic outcomes were satisfactory, the tolerance study warrants concern. Notably, sinus bradycardia observed in healthy young males between the ages of 22 and 25 post-administration might be benign in this population but could pose serious risks in elderly AD patients. These safety concerns may have contributed to the discontinuation of ZT-1 development. Although the exact reasons remain undisclosed, important lessons can be drawn for future AD drug development. In particular, BuChE inhibition and potential cardiotoxicity should be carefully evaluated during preclinical studies, including animal models, to mitigate adverse effects in patients with mild to moderate AD.

Excitingly, a recent study using fluorine-containing Schiff base derivatives of huperzine A was conducted [40]. This preclinical study aims to investigate the inhibition of Aβ aggregation through paralysis assays in CL4176 worms, SOD production, and the expression of anti-Aβ genes, *skn-1* and *daf-16*. These Schiff base derivatives target the underlying causes of AD rather than the symptomatic treatment approach of ZT-1 and showed promising results.

## 6. Future Perspectives

The growing body of evidence implicating neuroinflammation as a central pathological mechanism in AD has revolutionized our understanding of disease progression and therapeutic intervention. Over the past two decades, the search for effective therapeutics has expanded from amyloid- and tau-centric strategies to those targeting inflammatory and oxidative cascades. Among emerging candidates, Schiff base derivatives offer a unique advantage owing to their structural flexibility and multifunctional biological properties. However, several strategic advances are required to fully harness their therapeutic potential.

### 6.1. Targeting Multiple Pathways

The multifactorial etiology of AD necessitates therapeutics that can intervene at multiple pathological nodes simultaneously. Schiff bases, due to their versatile imine core, provide a valuable scaffold for the development of MTDLs capable of inhibiting AChE, chelating metal ions, suppressing oxidative stress, and modulating inflammatory responses. Future efforts must prioritize the design of hybrid molecules that can synergistically address interconnected pathways in AD, particularly Aβ aggregation, tau phosphorylation, and neuroinflammation. Hybrid drug development has gained attention for various diseases, and many candidates are currently at the preclinical and clinical stages. To date, tyrosine kinase inhibitors such as lapatinib (Tykerb) and sunitinib (Sutent) are Food and Drug Administration (FDA)-approved hybrid drugs. Lapatinib, used to treat breast cancer, inhibits the autophosphorylation of intracellular tyrosine kinase domains in human epidermal growth factor receptor type 2 (HER2/ERBB2) and epidermal growth factor receptor (HER1/EGFR/ERBB1) upon ligand binding. This halts the activation of the MAPK pathway, phosphatidylinositol 3-kinase/Akt (PI3K/Akt) pathway, and mammalian target of rapamycin (mTOR)-dependent transduction pathways, causing growth arrest or apoptosis of tumor cells [50]. Meanwhile, sunitinib is used for renal cell carcinoma treatment and inhibits various tyrosine kinase receptors such as platelet-derived growth factor receptors (PDGFRa and PDGFRb), vascular endothelial growth factor receptors (VEGFR1, VEGFR2 and VEGFR3), stem cell factor receptor (KIT), Fms-like tyrosine kinase-3 (FLT3), colony stimulating factor receptor Type 1 (CSF-1R), and the glial cell line-derived neurotrophic factor receptor (RET), which are involved in tumor growth, pathologic angiogenesis, and metastatic progression of cancer [51].

### 6.2. Nanocarrier-Based Drug Delivery Systems

One of the primary limitations of Schiff bases is their instability under physiological conditions and limited permeability across the BBB. The application of nanotechnology, particularly polymeric nanoparticles, liposomes, and dendrimer-based carriers, can markedly enhance the pharmacokinetic profile, CNS delivery, and bioavailability of Schiff base derivatives. For instance, the intravenous application of liposomes has shown to bypass efflux transporters at the BBB and significantly increase brain uptake in animal models [52]. Meanwhile, the fast-release, water-soluble galatamine hydrobromide used to treat AD exhibits delayed release when incorporated into chitosan-based nanoparticles. Moreover, the release of galantamine hydrobromide from chitosan-based nanoparticles was found to best fit the Higuchi model, indicating a diffusion-driven mechanism influenced by polymer swelling and gradual particle erosion [53]. Nanocarrier-based systems can also facilitate targeted drug release, minimizing peripheral toxicity and improving therapeutic outcomes in vivo.

### 6.3. Artificial Intelligence and SAR-Guided Design

Recent advances in artificial intelligence (AI) and machine learning are poised to revolutionize drug discovery. These tools can be leveraged to predict BBB permeability, optimize pharmacodynamics profiles, and refine structure–activity relationships (SARs) with unprecedented precision. AI-guided virtual screening and molecular docking simulations can expedite the identification of lead Schiff bases with desirable pharmacological and physicochemical attributes. Although studies have been conducted using this approach, exploration remains limited. For example, SAR and molecular docking analyses were consistent with in vitro anti-cholinesterase results, indicating that pyrrole-derived triazole-based Schiff bases substituted with a –CF_3_ group possess significant cholinesterase inhibitory potential. Furthermore, absorption, distribution, metabolism, excretion, and toxicity (ADMET) profiling confirmed that these analogues exhibit favorable drug-like properties and are predicted to be therapeutically safe [21].

### 6.4. Personalized and Precision Medicine Approaches

The advent of precision medicine in neurodegenerative disorders underscores the need to tailor therapeutic strategies to individual genetic and biochemical profiles. Schiff base–metal complexes, when paired with biomarker-based diagnostics, could be designed to target specific dysregulated metal ions such as copper, iron, and zinc implicated in patient-specific AD subtypes. The development of chelation therapies that selectively target pathological metal pools without perturbing physiological homeostasis represents a major research frontier [54,55].

### 6.5. Advancing Translational and Clinical Research

To date, most Schiff base derivatives remain confined to in vitro or early in vivo experimentation. The translation of these compounds into clinically viable drugs will require robust pharmacological evaluations, toxicity profiling, and behavioral assessments in higher-order animal models. Moreover, clinical trial frameworks should be developed to test the safety, efficacy, and cognitive outcomes of promising Schiff base-based therapeutics in human populations. Interdisciplinary collaboration across medicinal chemistry, pharmacology, and clinical neuroscience will be essential to bridge this translational gap.

## 7. Conclusions

AD remains an intractable neurodegenerative disorder characterized by progressive cognitive decline and complex multifactorial pathology. Neuroinflammation has emerged as a central pathological mechanism that not only exacerbates amyloid and tau pathology but also perpetuates neuronal dysfunction and loss. Targeting neuroinflammatory cascades offers a promising therapeutic avenue that complements traditional amyloid- and tau-focused strategies.

Schiff base derivatives, with their versatile imine scaffold, exhibit significant potential as multi-target agents capable of modulating key pathological features of AD, including inflammation, oxidative stress, ROS dysregulation, metal ion dyshomeostasis, Aβ accumulation, and cholinergic dysfunction. Furthermore, their neuroprotective and anti-amnesic profiles may prevent disease progression and enhance recovery. However, these activities have been investigated across diverse Schiff base structures, and systematic evaluation of metal-incorporated Schiff bases remains limited. Comprehensive studies assessing their multi-target potential alongside their pharmacokinetic profiles are therefore essential to advance their development as prospective anti-AD agents. Additionally, the translation of Schiff bases from bench to bedside faces considerable challenges, including stability, bioavailability, BBB permeability, and comprehensive in vivo validation.

Future research must emphasize rational design through SAR studies, leverage advanced drug delivery platforms, and embrace AI-driven discovery to optimize these compounds. Ultimately, integrated translational and clinical efforts will be essential to validate Schiff base derivatives as viable therapeutic candidates. With continued interdisciplinary collaboration, Schiff bases hold promise to enrich the therapeutic landscape of AD by addressing neuroinflammation as a pivotal driver of disease progression.

## Figures and Tables

**Table 1:** Recent Schiff base derivatives and their mechanistic targets in Alzheimer’s disease.

Compound Type	Reported Mechanism(s)	Level of Evidence	Key Findings/Outcomes	Reference(s)
Pyrimidine-based Schiff base complexes	Inhibition of amyloid-beta (Aβ) aggregation	Cell-based model	Reduced amyloid fibril formation in vitro	[25]
Sulfonate Schiff base	Carbonic anhydrases inhibition; acetylcholinesterase/butyrylcholinesterase (AChE/BuChE) inhibition	In vitro enzyme assays; molecular docking	Dual enzymatic inhibition with nanomolar I half-maximal inhibitory concentration (IC_50_) values	[26]
Mn(II), Co(II), Ni(II), Cu(II), Zn(II), VO(II) azo–Schiff base	Neuroprotective activity	Cell-based model	Improved neuronal viability	[22]
Schiff base-synthesized copper oxide nanoparticles	Antioxidant; free radical scavenging; AChE inhibition; anti-inflammatory	In vitro enzyme assays	Schiff base-synthesized copper oxide nanoparticles demonstrated superior in vitro biological activities when compared to the ligand	[27]
Pt and Ru Schiff base complexes	Inhibition of Aβ aggregation; neuroprotective	Cell-based model	Reduced β-sheet formation and protected neuronal cells	[28,29]
Vanillin-based Schiff bases	Antioxidant; AChE inhibition; antiamnesic in rodents	In vitro enzyme assays; in vivo animal behavioral studies	Improved memory performance in the scopolamine-induced amnesia model	[30]
Pyrrole-derived triazole Schiff base	AChE/BuChE inhibition	In vitro enzyme assays; molecular docking	Potent cholinesterase inhibition compared to the standard drug, Donepezil, and Allanzanthone	[21]
Aryl Schiff base	Carbonic anhydrases inhibition; AChE/BuChE inhibition	In vitro enzyme assays; molecular docking	Multi-target inhibition with selectivity toward hCA II	[31]
Schiff bases	Aβ inhibition in transgenic CL4176 *Caenorhabditis elegans*	In vivo animal studies	Delayed paralysis onset induced by Aβ expression in a transgenic nematode model	[32]
Pyrazole-based Schiff bases	Antioxidant; free radical scavenging; AChE inhibition; anti-inflammatory	In vitro enzyme assays	Suppressed neuroinflammation and oxidative markers in vitro	[33,34]
Benzo[d]oxazole bis–Schiff base	AChE/BuChE inhibition	In vitro enzyme assays; molecular docking	Strong dual cholinesterase inhibition with favorable selectivity toward BuChE	[35]
Benzimidazole-based Schiff base	AChE/BuChE inhibition	In vitro enzyme assays; molecular docking	Three compounds outperform the standard drug, Donepezil	[36]
Amino acid Schiff base Zn(II) complexes	AChE/BuChE inhibition	In vitro enzyme assays; molecular docking	High selectivity for BuChE over AChE	[37]
2-Mercaptobenzimidazole hydrazone Schiff base derivatives	AChE/BuChE inhibition; Ca^2+^ antagonistic activity	In vitro enzyme assays; molecular docking	Suppressed AChE and modulated intracellular calcium signaling	[38]
Lamellarin-like Schiff bases	Inhibition of Aβ aggregation; AChE/BuChE inhibition; MAO inhibition	In vitro enzyme assays	Multi-target profile including both anti-Alzheimer and antidepressant	[39]
Fluorine-containing Schiff-base derivatives of huperzine A	Aβ inhibition in transgenic CL4176 *Caenorhabditis elegans*	In vivo animal studies; molecular docking	Higher % of non-paralyzed transgenic nematode model; Higher superoxide dismutase (SOD) production and expression of *Skn-1* and *daf-16*	[40]

## Data Availability

No new data were created or analyzed in this study. Data sharing is not applicable to this article.

## Abbreviations

The following abbreviations are used in this manuscript:

AChE	acetylcholinesterase
AD	Alzheimer’s disease
ADAS-cog	assessment cognitive subscale
ADMET	absorption, distribution, metabolism, excretion, and toxicity
AI	artificial intelligence
APP	amyloid precursor protein
Aβ	amyloid-beta
BBB	blood-brain barrier
BChE	butyrylcholinesterase
BRAINz	Better Recollection for Alzheimer’s patients with ImplaNts of ZT-1
CNS	central nervous system
CSF-1R	Colony-stimulating factor receptor Type 1
DAM	disease-associated microglia
FDA	Food and Drug Administration
FLT3	Fms-like tyrosine kinase-3
HAT	hydrogen atom transfer
HER1/EGFR/ERBB1	epidermal growth factor receptor
HER2/ERBB2	human epidermal growth factor receptor type 2
IL-10	interleukin-10
IL-1β	interleukin-1β
IL-6	interleukin-6
JAK/STAT	Janus kinase/signal transducer and activator of transcription
KIT	stem cell factor receptor
MAPK	mitogen-activated protein kinase
MMSE	Mini-Mental State Examination
MTDLs	multi-target-directed ligands
mTOR	mammalian target of rapamycin
NF-κB	nuclear factor kappa-light-chain-enhancer of activated B cells
NLRs	NOD-like receptors
NO	nitric oxide
NOD	nucleotide-binding oligomerization domain
NORT	new object recognition test
NPI-Q	Neuropsychiatric Inventory Questionnaire
PDGFR	platelet-derived growth factor receptors
PI3K/Akt	phosphatidylinositol 3-kinase/Akt
PRR	pattern recognition receptors
RAGE	receptors for advanced glycoxidation end-products
RET	glial cell-line derived neurotrophic factor receptor
RNS	reactive nitrogen species
ROS	reactive oxygen species
SARs	structure–activity relationships
SET	single electron transfer
TLR	Toll-like receptors
TNF-α	tumor necrosis factor-alpha
TREM2	triggering receptor expressed on myeloid cells 2
VEGFR	vascular endothelial growth factor receptor

## References

[1] Gauthier S., Webster C., Servaes S., Morais J.A., Rosa-Neto P. (2022). World Alzheimer Report 2022: Life After Diagnosis—Navigating Treatment, Care and Support.

[2] Cummings J., Lee G., Ritter A., Sabbagh M., Zhong K. (2019). Alzheimer’s disease drug development pipeline: 2019. Alzheimer’s Dement..

[3] Heneka M.T., Carson M.J., El Khoury J., Landreth G.E., Brosseron F., Feinstein D.L., Jacobs A.H., Wyss-Coray T., Vitorica J., Ransohoff R.M. (2015). Neuroinflammation in Alzheimer’s disease. Lancet Neurol..

[4] Ransohoff R.M. (2016). How neuroinflammation contributes to neurodegeneration. Science.

[5] Leng F., Edison P. (2021). Neuroinflammation and microglial activation in Alzheimer disease: Where do we go from here?. Nat. Rev. Neurol..

[6] Thakur S., Jaryal A., Bhalla A. (2024). Recent advances in biological and medicinal profile of Schiff bases and their metal complexes: An updated version (2018–2023). Results Chem..

[7] Jia J.-Y., Zhao Q.-H., Liu Y., Gui Y.-Z., Liu G.-Y., Zhu D.-Y., Yu C., Hong Z. (2013). Phase I study on pharmacokinetics and tolerance of ZT-1, a prodrug of huperzine A, for treatment of Alzheimer’s disease. Acta Pharmacol. Sin..

[8] Leman L., Kitson S.L., Brown R.T., Cairns J., Watters W., McMordie A., Murrell V.L., Marfurt J. (2011). Synthesis of isotopically labelled [14C]ZT-1 (Debio-9902), [d3]ZT-1 and (−)-[d3]huperzine A. J. Label. Compd. Radiopharm..

[9] Bilbo S., Stevens B. (2017). Microglia: The brain’s first responders. Cerebrum.

[10] Heneka M.T., Kummer M.P., Latz E. (2014). Innate immune activation in neurodegenerative disease. Nat. Rev. Immunol..

[11] Hansen D.V., Hanson J.E., Sheng M. (2018). Microglia in Alzheimer’s disease. J. Cell Biol..

[12] Singh D. (2022). Astrocytic and microglial cells as modulators of neuroinflammation in Alzheimer’s disease. J. Neuroinflamm..

[13] Rojo L.E., Fernandez J.A., Maccioni A.A., Jimenez J.M., Maccioni R.B. (2008). Neuroinflammation in pathogenesis and molecular diagnosis of Alzheimer’s disease. Arch. Med. Res..

[14] Liddelow S.A., Barres B.A. (2017). Reactive astrocytes: Production, function, and therapeutic potential. Immunity.

[15] Azizi G., Khannazer N., Mirshafiey A. (2014). Potential role of chemokines in Alzheimer’s disease pathogenesis. Am. J. Alzheimer’s Dis. Other Dement..

[16] Twarowski B., Herbet M. (2023). Inflammatory processes in Alzheimer’s disease—Pathomechanism, diagnosis and treatment: A review. Int. J. Mol. Sci..

[17] Da Silva C.M., Da Silva D.L., Modolo L.V., Alves R.B., De Resende M.A., Martins C.V.B., De Fátima Á. (2011). Schiff bases: A short review of their antimicrobial activities. J. Adv. Res..

[18] Mumtaz A., Mahmud T., Khalid M., Khan H., Sadia A., Samra M.M., Basra M.A.R. (2022). Biological evaluation of synthesized Schiff base–metal complexes derived from sulfisomidine. J. Pharm. Innov..

[19] Chohan Z.H., Arif M., Akhtar M.A., Supuran C.T. (2006). Metal-based antibacterial and antifungal agents: Co(II), Cu(II), Ni(II) and Zn(II) complexes with amino acid–derived compounds. Bioinorg. Chem. Appl..

[20] Barnham K.J., Masters C.L., Bush A.I. (2004). Neurodegenerative diseases and oxidative stress. Nat. Rev. Drug Discov..

[21] Khan S., Iqbal T., Khan M.B., Hussain R., Khan Y., Darwish H.W. (2024). Novel pyrrole-based triazole moiety as therapeutic hybrid: Anti-Alzheimer potential with molecular mechanism. BMC Chem..

[22] Sarwade K.N., Sakhare K.B., Sakhare M.A., Thakur S.V. (2025). Neuroprotective, anticancer and antimicrobial activities of azo-Schiff base ligand and its metal complexes. Eurasian J. Chem..

[23] Boulguemh I.-E., Beghidja A., Khattabi L., Long J., Beghidja C. (2020). Copper(II) complexes based on a thiophene carbohydrazide Schiff base ligand. Inorg. Chim. Acta.

[24] Savcı A., Turan N., Buldurun K., Eşref Alkış M., Alan Y. (2022). Schiff base containing fluorouracil and its M(II) complexes: Cytotoxic and antioxidant activities. Inorg. Chem. Commun..

[25] Irisli S., Cakir A., Gunnaz S. (2025). Schiff base complexes targeting amyloid-β aggregation in Alzheimer’s disease. Bioorg. Chem..

[26] Yasar U., Demir Y., Gonul I., Ozaslan M.S., Celik G.G., Turkes C., Beydemir S. (2025). Schiff base sulfonate derivatives as carbonic anhydrase and acetylcholinesterase inhibitors. Chem. Biodivers..

[27] Almehizia A.A., Naglah A.M., Aljafen S.S., Hassan A.S., Aboulthana W.M. (2025). Biological activities of Schiff base–synthesized copper oxide nanoparticles. Pharmaceutics.

[28] Gunnaz S., Yildiz E., Tuncel Oral A., Yurt F., Erdem A., Irisli S. (2025). Schiff base–platinum and ruthenium complexes with anti-Alzheimer properties. J. Inorg. Biochem..

[29] Irisli S., Günnaz S., Özcan O., Ari A., Maral M., Erdem A., Özel D., Yurt F. (2024). Platinum(II) Schiff base complexes inhibiting amyloid β1–42 aggregation. Appl. Organomet. Chem..

[30] Gul Q., Karim N., Shoaib M., Zahoor M., Rahman M.U., Bilal H., Ullah R., Alotaibi A. (2024). Vanillin derivatives as antiamnesic agents. Heliyon.

[31] Ozil M., Balaydin H.T., Dogan B., Senturk M., Durdagi S. (2024). Aryl Schiff base derivatives as inhibitors of hCA I, hCA II, AChE, and BuChE. Arch. Pharm..

[32] Thakor P.M., Patel J.D., Patel R.J., Chaki S.H., Khimani A.J., Vaidya Y.H., Chauhan A.P., Dholakia A.B., Patel V.C., Patel A.J. (2024). New Schiff bases: Synthesis, characterization, biomedical applications. ACS Omega.

[33] Naglah A., Almehizia A., Al-Wasidi A., Alharbi A., Alqarni M., Hassan A., Aboulthana W. (2024). Pyrazole-based Schiff bases as multi-target anti-Alzheimer agents. Pharmaceuticals.

[34] Alkahtani H.M., Almehizia A.A., Al-Omar M.A., Obaidullah A.J., Zen A.A., Hassan A.S., Aboulthana W.M. (2023). Schiff bases bearing pyrazole scaffold as antioxidant, anti-diabetic, anti-Alzheimer agents. Molecules.

[35] Taha M., Rahim F., Zaman K., Anouar E., Uddin N., Nawaz F., Sajid M., Khan K., Shah A., Wadood A. (2023). Benzo-d-oxazole bis Schiff bases as anti-Alzheimer agents. J. Biomol. Struct. Dyn..

[36] Hussain R., Khan S., Ullah H., Ali F., Khan Y., Sardar A., Iqbal R., Ataya F.S., El-Sabbagh N.M., Batiha G.E. (2023). Benzimidazole-based Schiff base hybrids for multi-target Alzheimer drug development. Pharmaceuticals.

[37] Senocak A., Tas N.A., Taslimi P., Tuzun B., Aydin A., Karadag A. (2022). Amino acid Schiff base Zn(II) complexes as therapeutic approaches in diabetes and Alzheimer’s disease. J. Biochem. Mol. Toxicol..

[38] Begum F., Rehman N., Khan A., Iqbal S., Paracha R., Uddin J., Al-Harrasi A., Lodhi M. (2022). Hydrazone Schiff bases for Alzheimer’s therapy. Front. Pharmacol..

[39] Nevskaya A., Anikina L., Purgatorio R., Catto M., Nicolotti O., de Candia M., Pisani L., Borisova T., Miftyakhova A., Varlamov A. (2021). Homobivalent lamellarin-like Schiff bases. Molecules.

[40] Wang R.-Y., Huang J.-S., Gu X.-L., Tan W.-W., Cui H., Yang B.-C., Yang T. (2026). Fluorine-containing Schiff-base derivatives of huperzine A. J. Mol. Struct..

[41] Shareef S.H. (2024). Gastroprotective effects of Schiff base CdCl_2_ compound. Cureus.

[42] Zahedifard M., Faraj F., Paydar J., Looi C.Y., Hajrezaei M., Hasanpourghadi M., Kamalidehghan B., Abdul Majid N., Ali H., Abdulla M. (2015). Quinazolinone Schiff bases inducing apoptosis in MCF-7 cells. Sci. Rep..

[43] Rana M.S., Rayhan N.M.A., Emon M.S.H., Islam M.T., Rathry K., Hasan M.M., Islam Mansur M.M., Srijon B.C., Islam M.S., Ray A. (2024). Antioxidant activity of Schiff base ligands: Review. RSC Adv..

[44] Anouar H., Raweh S., Bayach I., Taha M., Baharudin M.S., Di Meo F., Hasan M.H., Adam A., Ismail N.H., Weber J.F. (2013). Antioxidant properties of phenolic Schiff bases. J. Comput. Aided Mol. Des..

[45] Tang Y.Z., Liu Z.Q. (2007). Free-radical-scavenging mechanism of hydroxyl-substituent Schiff bases. Cell Biochem. Funct..

[46] Mostafa M.A., Ismail M.M., Morsy J.M., Hassanin H.M., Abdelrazek M.M. (2023). Chitosan Schiff bases with anticancer and antioxidant activities. Polym. Bull..

[47] Kumar A., Sidhu J., Lui F., Tsao J.W. (2025). Alzheimer Disease. StatPearls.

[48] Li C., Du F., Yu C., Xu X., Zheng J., Xu F., Zhu D. (2004). Determination of ZT-1 and huperzine A in rat blood. Rapid Commun. Mass Spectrom..

[49] Debiopharm Group Clinical Update—Debio 9902 (ZT-1) for Alzheimer’s Disease. https://www.debiopharm.com/drug-development/press-releases/clinical-update-debio-9902-zt-1-for-alzheimers-disease/.

[50] Opdam F.L., Guchelaar H.J., Beijnen J.H., Schellens J.H. (2012). Lapatinib for breast cancer. Oncologist.

[51] Schmid T.A., Gore M.E. (2016). Sunitinib for metastatic renal cell carcinoma. Ther. Adv. Urol..

[52] Helm F., Fricker G. (2015). Liposomal conjugates for CNS drug delivery. Pharmaceutics.

[53] Georgieva D., Nikolova D., Vassileva E., Kostova B. (2023). Chitosan nanoparticles for nasal galantamine delivery. Pharmaceutics.

[54] Gong L., Zhang X., Ge K., Yin Y., Machuki J.O., Yang Y., Shi H., Geng D., Gao F. (2021). Carbon nitride-based nanocaptor for Alzheimer’s phototherapy. Biomaterials.

[55] Li D.D., Zhang W., Wang Z.Y., Zhao P. (2017). Serum copper, zinc, and iron in Alzheimer’s disease: Meta-analysis. Front. Aging Neurosci..

